# The combined association of dietary inflammatory index and resting metabolic rate on cardiorespiratory fitness in adults

**DOI:** 10.1186/s41043-023-00413-2

**Published:** 2023-07-13

**Authors:** Hossein Shahinfar, Nastaran Payandeh, Kimia Torabynasab, Mahshid Shahavandi, Saba Mohammadpour, Nadia Babaei, Mojdeh Ebaditabar, Kurosh Djafarian, Sakineh Shab-Bidar

**Affiliations:** 1 Department of Nutrition, School of Public Health, Iran University of Medical Sciences, Tehran, Iran; 2 Department of Community Nutrition, School of Nutritional Sciences and Dietetics, Tehran University of Medical Sciences (TUMS), No 44, Hojjat-dost Alley, Naderi St., Keshavarz Blvd, Tehran, Iran; 3 Department of Clinical Nutrition and Dietetics, Faculty of Nutrition Science and Food Technology, Shahid Beheshti University of Medical Sciences, Tehran, Iran; 4 Department of Clinical Nutrition, School of Nutritional Sciences and Dietetics, Tehran University of Medical Sciences, Tehran, Iran

**Keywords:** Dietary inflammatory index, Combined association, Resting metabolic rate, Cardiorespiratory fitness, Interaction

## Abstract

**Background:**

No study has examined the combined association of dietary inflammatory index (DII) of the diet and resting metabolic rate (RMR) on cardiorespiratory fitness (CRF). Therefore, we investigated the combined association between DII and RMR on CRF.

**Methods:**

This cross-sectional study was conducted on 270 adult subjects. The DII was calculated using a validated semi-quantified food frequency questionnaire. RMR was measured using an indirect calorimetric method. Socioeconomic status, anthropometric measures, body composition and blood pressure were documented by a trained interviewer. CRF was assessed by using Bruce protocol. Binary logistic regression was performed to find the association of CRF with DII/RMR categories in various models.

**Results:**

The participants categorized into four groups including: (1) low DII/high RMR, (2) low DII/low RMR, (3) high DII/low RMR, (4) high DII/high RMR. The mean of VO_2Max_ (mL/kg/min), VO_2max_ (L/min) and VO_2max_ relative to lean body mass (LBM) was lower in participants that were classified as high DII/low RMR compared to those in low DII/high RMR. After controlling for age, sex, education status, smoking status, and physical activity those who were in the high DII/low RMR group, compared to the low DII/high RMR group were 28% less likely to have higher VO_2max_ (ml/kg/min) (OR 0.72; 95% CI 0.18, 0.82, *p *= 0.04). Moreover, had 25% lower odds of VO_2max_ (L/min) which was significant (OR 0.75, 95% CI 0.11, 0.89, *p *= 0.03). In addition, were 21% less likely to have higher VO_2max_ (LBM) (OR 0.79; 95% CI 0.30, 0.92, *p *= 0.02).

**Conclusions:**

Overall, consumption of a pro-inflammatory diet in combination with low RMR status is associated with lower odds of CRF compared to those who had anti-inflammatory diet in combination with high RMR status among Iranian healthy adults. This study suggests that researchers should focus on combined relationships rather than single pair-wise associations for having a better judgment.

## Introduction

Resting metabolic rate (RMR) is the least energy needed to keep up essential body function during a stable resting state and fasting status [1]. It is estimated that lean body mass is accounts for 60–85% of RMR [2]. Previous studies have shown significant inverse association between RMR and body fat mass and body weight, such that decreasing in RMR may resulted in increasing body fat mass and body weight [3, 4]. Additionally, inflammation may play a role in weight gain through leptin and insulin resistance leading to increased FM and a balance between energy intake and expenditure [5, 6]. Indeed, increased body fat mass in obese individuals results in increasing C-reactive protein (CRP) and inflammatory cytokines [7]. Therefore, obesity is considered a low-grade inflammatory condition [8]. Besides, this chronic inflammation in adipose tissue accelerates the complications and diseases caused by obesity [9]. The study results showed that there was a positive relationship between C-reactive protein synthesis index (CRP) and the risk of coronary heart disease and mortality from cardiovascular disease [10, 11]. Accumulating evidence also suggests that obesity reduces cardiorespiratory fitness (CRF) [12]. CRF is a modifiable and independent risk factor for mortality from cardiovascular disease (CVD) [13]. Previous studies have shown that high CRF, which is evaluated by the peak of oxygen uptake (VO_2Max_), is associated with a reduced risk of cardiovascular disease and related mortality [14]. Therefore, inflammation and VO_2Max_ are significantly associated with other major cardiovascular risk factors [14]. One of the key and modifiable factors effective in reducing or causing inflammation is diet which led to the development of the dietary inflammatory index (DII) [15]. In fact, DII is a scoring algorithm that ranks individuals' diets based on their inflammatory potential [16]. The purpose of making this index is to classify people’s diet from maximally anti-inflammatory to maximally pro-inflammatory [15–18]. The DII authors evaluated the association of dietary components with six markers of inflammation:

IL-1*β*, IL-4, IL-6, IL-10, TNF-*α* and CRP [16]. Accumulating evidence illustrate that a high DII diet is associated with an increased risk of metabolic syndrome, diabetes, hypertension, and cancer [7, 12, 13, 19]. In addition, a recent umbrella review showed that anti-inflammatory dietary patterns play a significant role in the prevention of chronic diseases [20].

Given that Iran has an increasing rate of obesity and several inflammatory diseases, we designed this cross-sectional study to investigate whether the combined association of dietary inflammatory index and resting metabolic rate is related to cardiorespiratory fitness in adults. We hypothesized that the higher inflammatory index of the diet in our participants is associated with low RMR and CRF in Iranian adults.

## Methods

### Study design

**Fig. 1:**
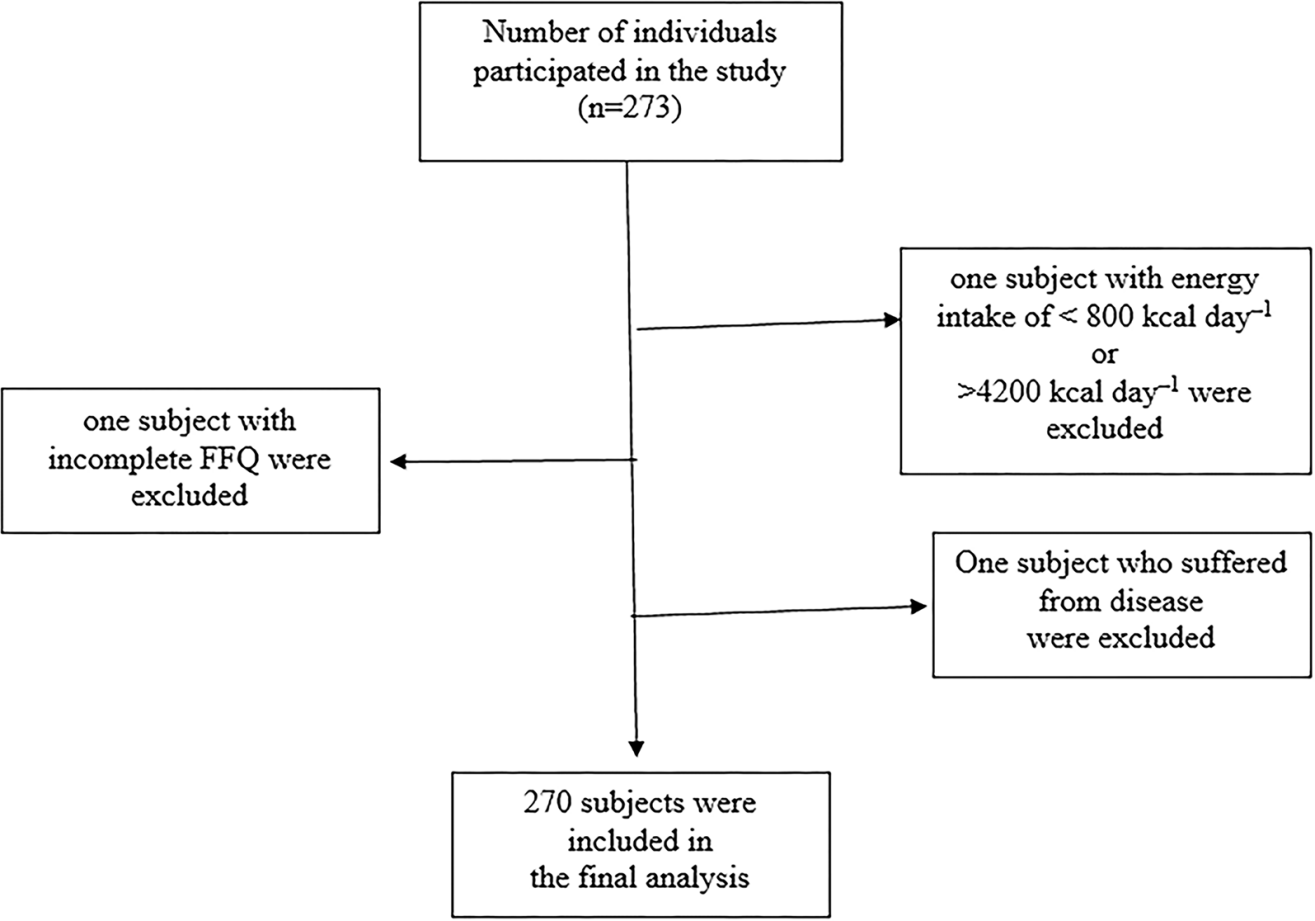
Participants flow diagram

This study consisted of 270 apparently healthy adults (118 men and 152 women). The social network was used to recruit participants through a recruitment message. Convenience sampling was used to select the subjects. Based on previously calculated correlation coefficient between diet and cardiorespiratory fitness [21], our target number of participants was 256 $\left( {\left( {{{Z_{{1 - \frac{\alpha }{2}}} + Z_{1 - \beta } \times \sqrt {1 - r^{2} } } \mathord{\left/ {\vphantom {{Z_{{1 - \frac{\alpha }{2}}} + Z_{1 - \beta } \times \sqrt {1 - r^{2} } } r}} \right. \kern-0pt} r}} \right) = 256} \right)$. However, in order to replace patients who were excluded due to under- or over-reported food intakes, we continued sampling until enrolling 273 individuals. Research criteria included apparently healthy adults living in Tehran, aged 18–70, who were interested in participating in the study, and were willing to participate in study. Individuals with extreme values of dietary intake (less than 800 kcal per day or more than 4200 kcal per day, respectively), those with kidney, liver, digestive, hormonal and lung disease, infectious and active inflammatory diseases, pregnancy, lactation, routine supplement and drug use, such as weight loss, hormonal, sedative drugs, thermogenic supplements such as caffeine and green tea and conjugated linoleic acid (CLA), were excluded. After removing three subjects due to above-mentioned reasons, only 270 participants remained for statistical analysis (Fig. 1).

### Anthropometric measures

A wall stadiometer was used to determine the height of participants without shoes (Seca, Germany). Waist circumference (WC) was measured at narrowest point between lower rib and iliac crest by non-elastic tape. Body mass index (BMI), weight, fat mass (FM), fat free mass (FFM), and lean body mass (LBM) were measured by InBody (InBody720, Biospace, Tokyo, Japan). The established protocol entailed abstaining from food consumption for a minimum of 4 h, consuming at least 2 L of water the day prior, and refraining from consuming coffee or alcoholic beverages for a minimum of 12 h. Prior to the test, participants were instructed to void their bladder [22].

### Assessment of other variables

The participants filled out a self-administered questionnaire to assess their demographics, including their age, sex, smoking status (smokers, non-smokers or quitters), as well as their education status (under diploma/diploma/educated). To assess blood pressure, first, we demanded individuals to rest for at least ten minutes. Blood pressure was then measured using a standard mercury sphygmomanometer, twice with a 5-min interval, while participants were sitting. The mean of the two measurements was recorded as the participant’s blood pressure. The levels of physical activity were measured using the international physical activity questionnaire (IPAQ) [23]. Three categories were developed to categorize the subjects, including very low (< 600 METs/week), low (600–3000 METs/week), moderate, and high (> 3000 METs/week) based on metabolic equivalents (METs) [24].

### Dietary intakes

In order to evaluate habitual food consumption, a validated semi-quantitative food frequency questionnaire were used [25]. The questionnaire included 168 food items, with standard serving sizes as commonly consumed by Iranians. A team of experienced nutritionists interviewed each participant in detail to collect nutritional information. Participants were queried on their consumption of various food items, with two questions posed for each item: Firstly, the frequency of food group consumption, measured in annual, monthly, weekly, and daily intervals over the past year, and secondly, the approximate amount of each item consumed per occasion. Subsequently, all food items' frequency and quantity of consumption were converted into grams per day, utilizing “household measures” [26]. The authors added Iranian foods and recipes to the software and the macronutrient and micronutrient content of the diets were then determined using modified Nutritionist IV software developed specifically for Iranian foods (version 7.0; N-Squared Computing, Salem, OR, USA).

### Resting metabolic rate

The resting metabolic rate (RMR) was estimated through indirect calorimetry (Cortex Metalyser 3B, Leipzig, Germany). As per established procedures, two calibrations were undertaken: (1) The gas analyzer was calibrated prior to each measurement using ambient air and a standard gas mixture (16% O_2_, 4.96% CO_2_), and (2) the flow calibration was executed via a 3-L syringe (Hans Rudolph, UK). Upon completion of the calibration process, data pertaining to the patient's date of birth, sex, height, weight, and mask size were entered. Patients were instructed to abstain from food and non-water fluids for 12 h and refrain from smoking for a minimum of 4 h prior to the test. Participants were provided with guidelines to remain alert and relaxed while positioned supine on a bench, and refrain from talking or moving during the examination. The measurement was conducted within a serene environment with controlled temperature and humidity, lasting for 45 min after donning a gas collection mask. Readings were taken without interruption, and the first 10 min were excluded from the data analysis [27].

### Cardiorespiratory fitness testing

**Fig. 2:**
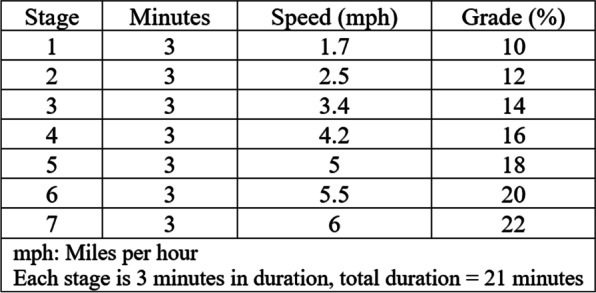
Bruce protocol for maximal and sub maximal efforts

To estimate cardiorespiratory fitness (CRF), study participants commenced their exercise regimen at a velocity of 5 miles per hour (mph) for a duration of 5 min, employing a standard treadmill model (h/p/cosmos). VO_2max_ was evaluated through the implementation of the Bruce Protocol [28], which is systematically structured into incremental 3-min stages that initiate at a pace of 1.7 mph and an incline of 10% gradient for 3 min, subsequently advancing in stages (Fig. 2) until a stop-test indicator is attained. This protocol consisted of seven stages that each stage last 3 min. The test is halted if the patient experiences chest pain, shortness of breath or fatigue. The test is also terminated if more than 90% of maximum heart rate predicted for age is reached, respiratory exchange ratio is ≥ 1.10, and a plateau (< 150 mL/min increase) in oxygen consumption is detected in contrast with an increase in speed. At least two of the three criteria must be met. Finally, participants engage in a cool-down process consisting of a 3-min walk at 4 mph and stretching exercises. Following the Bruce protocol, the treadmill and respiration gas analyzer (Cortex Metabolizer 3B) were used to measure the three type of maximum oxygen consumption including relative to body mass [VO_2max_(ml/kg/min)], absolute [VO_2max_(L/min)], and relative to LBM [VO_2max_(LBM)].

### DII development

Dietary inflammatory index scores were calculated by multiplying 29-item nutrients or foods based on the inflammatory weights they carry according to Shivappa et al. method [16]. Firstly, in order to reduce the variation in dietary intake between people, macronutrients and micronutrients (carbohydrate, protein, total fat, cholesterol, saturated fatty acids, monounsaturated fatty acids (MUFA), polyunsaturated fatty acids (PUFA), n-3 fatty acids, n-6 fatty acids, *β*-carotene, vitamin A, vitamin C, vitamin D, vitamin E, vitaminB6, vitaminB12, fiber, folic acid, niacin, riboflavin, thiamin, iron, zinc, selenium, magnesium, onion, caffeine) were computed on a daily basis [29]; our documents lacked some nutrients (trans FAs, flavan-3-ol, flavones, flavonols, flavanones, anthocyanidins, isoflavones, pepper, thyme/oregano, rosemary, garlic, ginger, saffron, and turmeric and tea), so we excluded them. Food parameters were adjusted to their corresponding global mean and standard deviation for each individual [16]. To normalize the scoring system and avoid skewness, the *Z* score values were converted to percentiles and centered by doubling them and subtracting one. DII scores for food parameters are derived by multiplying the centered percentile value of each food parameter by the overall food parameter score [16]. Lastly, all food parameter-specific DII scores were summed to determine the DII score. Diets with higher DII scores tend to be pro-inflammatory, while diets with lower DII scores tend to be anti-inflammatory. We set median value of DII score as threshold level (DII median = 7.70) such that upper median values of DII considered as high inflammatory diet and vice versa.

### Statistical analysis

Statistical tests such as Kolmogorov–Smirnov and Shapiro–Wilk were used to determine the normality of distributions. There was a normal distribution for all variables. After that, subjects were categorized according to their DII and RMR median values. We computed four independent groups by combining DII and RMR dichotomized groups (low DII/low RMR, low DII/high RMR, high DII/low RMR and high DII/high RMR). To compare general characteristics across the four groups, we used one-way analysis of variance (ANOVA) and chi-square tests for quantitative and qualitative variables, respectively. To compare participants’ dietary intakes within four groups, analysis of covariance (ANCOVA) to adjust for energy intake. We used ANOVA to examine significant differences across the four above-mentioned groups. Post hoc Tukey test was used to compare pair-wise mean differences. CRF values were then transformed into binary variables according to the upper and lower median values. The median values were VO_2max_(ml/kg/min) = 30.0_,_ VO_2max_(L/min) = 2.04, and VO_2max_(LBM) = 47.94. ANCOVA test was performed to compare the mean of CRF among DII/RMR groups after adjusting for potential confounders such as age, sex, smoking status, energy intake, physical activity and BMI. Binary logistic regression was performed to find the association of CRF with DII/RMR categories in various models. First, we adjusted age and sex. Then, we additionally controlled for smoking and physical activity status. To obtain the overall trend of odds ratios across the combined effect of DII and RMR, we considered these classifications as an ordinal variable in the logistic regression models and the first tertiles regarded as the reference group. All statistical analysis was performed with the SPSS (Statistical Package for Social Sciences) for Windows 25.0 software package (SPSS, Chicago, IL). The level of statistical significance was pre-set at *p *< 0.05.

## Results

The general characteristics of participants are shown in Table 1. This research included a total of 270 participants (118 men and 152 women) with an age range of 18–70 years old. The mean of age, height, weight, BMI, WC, FFM and systolic blood pressure (SBP) had significant differences across study groups. For other variables, we did not see any significant difference. The distribution of sex among the four groups was significantly different.

**Table 1:** General characteristics of the participants in the study

	Low DII/low RMR (1)	Low DII/high RMR (2)	High DII/low RMR (3)	High DII/high RMR (4)	*P* value	Comparison*	Post hoc *P* value
*n*	67	67	68	68			
*Mean* ± *SD*							
Age (year)	39.8 ± 12.5	35.4 ± 12.7	39.3 ± 14.8	32.4 ± 11.6	0.004	(1) versus (4) (3) versus (4)	0.007 0.01
Height (cm)	163 ± 9.29	173 ± 9.15	162 ± 7.85	172 ± 9.06	< 0.001	(1) versus (2) (1) versus (4)	< 0.001 < 0.001
Weight (Kg)	67.8 ± 13.8	79.3 ± 14.3	64.4 ± 12.3	77.6 ± 17.8	< 0.001	(1) versus (2) (1) versus (4)	< 0.001 0.001
BMI (kg/m^2^)	25.1 ± 4.21	26.5 ± 5.00	24.2 ± 4.03	26.1 ± 5.22	0.022	(2) versus (3)	0.02
WC (cm)	87.8 ± 10.8	93.5 ± 12.5	84.1 ± 10.0	91.8 ± 14.2	< 0.001	(1) versus (2) (3) versus (4)	0.04 0.002
FFM (kg)	45.2 ± 10.3	55.1 ± 12.6	43.3 ± 9.43	55.1 ± 12.4	< 0.001	(1) versus (2) (1) versus (4)	< 0.001 < 0.001
FM (kg)	22.5 ± 7.57	23.56 ± 11.3	21.0 ± 7.24	22.6 ± 10.9	0.526	–	–
LBM (kg)	47.1 ± 9.74	46.2 ± 11.0	45.3 ± 10.6	49.0 ± 12.9	0.299	–	–
SBP (mmHg)	109 ± 19.5	118 ± 12.6	109 ± 18.1	107 ± 24.0	0.007	(1) versus (2) (2) versus (4)	0.04 0.006
DBP (mmHg)	70.3 ± 8.26	73.5 ± 10.5	69.1 ± 14.5	69.6 ± 8.77	0.102	–	–
*Frequency (%)*							
Sex, *n* (%)							
Male	12 (11.3)	38 (35.8)	16 (15.1)	40 (37.7)	< 0.001		
Female	51 (34.7)	25 (17)	47 (32)	24 (16.3)			
Smoking, *n* (%)							
Not smoking	58 (26.1)	53 (23.9)	56 (25.2)	55 (24.8)	0.231		
Quit smoking	1 (8.3)	6 (50)	4 (33.3)	1 (8.3)			
Smoking	9 (25)	5 (12.5)	5 (12.5)	18 (50)			
Physical activity, *n* (%)							
Low	26 (26.3)	17 (17.2)	28 (28.3)	28 (28.3)	0.238		
Moderate	26 (25.2)	27 (26.2)	24 (23.3)	26 (25.2)			
High	10 (20)	19 (38)	11 (22)	10 (20)			
Education, *n* (%)							
Under diploma	7 (16.7)	8 (22.2)	15 (38.9)	8 (22.2)	0.241		
Diploma	18 (40)	9 (20)	8 (17.8)	10 (22.2)			
Educated	41 (21.8)	49 (26.1)	48 (25.5)	50 (26.6)			

*P* value less than 0.05 was considered significant

Values are based on mean ± standard deviation or reported percentage

One-way ANOVA for quantitative data and chi-square test for qualitative data have been used

*DII* Dietary inflammatory index, *RMR* Resting metabolic rate, *WC* Waist circumference, *FFM* Fat free mass, *FM* Fat mass, *BMI* Body mass index, *LBM* Lean body mass, *mmHg* Millimeter of mercury, *Kg* Kilogram, *kg/m*^*2*^ Kilogram per meter^2^, *SBP* Systolic blood pressure, *DBP* Diastolic blood pressure

*Only significant comparisons are shown

Table 2 indicates the dietary intake of study participants by DII/RMR categories. There were significant differences in intake of protein, fiber, energy, vitamins (B12, B6, C) and total cholesterol between DII/RMR groups. Other dietary intakes had no significant differences.

**Table 2:** Dietary intake of study participants

	Low DII/low RMR	Low DII/high RMR	High DII/low RMR	High DII/high RMR	*P*_value_†
Participants, (*n*)	67	67	68	68	
Carbohydrate, g/d	306 ± 114	376 ± 152	327 ± 129	319 ± 163	0.151
Protein, g/d	78.8 ± 28.9	104 ± 44.2	80.1 ± 28.8	89.1 ± 38.6	0.017
Fat, g/d	71.5 ± 29.9	84.8 ± 34.4	71.7 ± 31.5	77.6 ± 29.6	0.752
Fiber, g/d	15.1 ± 6.67	18.2 ± 7.60	15.9 ± 7.32	13.7 ± 4.96	0.007
Energy†, Kcal/d	2134 ± 689	2634 ± 969	2222 ± 772	2289 ± 929	0.007
Vitamin B12, µg/d	3.72 ± 1.98	5.49 ± 3.46	3.93 ± 2.23	4.66 ± 2.25	0.025
Vitamin B6, mg/d	1.34 ± 0.64	1.73 ± 0.75	1.36 ± 0.65	1.32 ± 0.49	0.044
Vitamin A, µg/d	1249 ± 803	1624 ± 1261	1313 ± 782	1177 ± 815	0.272
Vitamin C, mg/d	138 ± 69.4	152 ± 78.2	140 ± 86.5	114 ± 47.5	0.032
Vitamin D, IU/d	1.81 ± 1.41	2.50 ± 2.20	2.27 ± 2.19	2.47 ± 2.46	0.477
Vitamin E, mg/d	3.95 ± 2.48	4.97 ± 3.01	4.49 ± 4.50	3.91 ± 1.48	0.522
Beta-carotene, µg/d	771 ± 689	1011 ± 1179	771 ± 659	660 ± 754	0.340
Caffeine, g/d	156 ± 129	269 ± 753	161 ± 116	179 ± 132	0.820
Total cholesterol, mg/d	254 ± 181	381 ± 280	233 ± 99.2	267 ± 131	0.003
Folate, mg/d	282 ± 117	345 ± 156	301 ± 129	293 ± 125	0.776
Iron, mg/d	19.7 ± 11.1	25.3 ± 12.8	19.9 ± 7.70	20.2 ± 10.3	0.458
Zinc, mg/d	8.52 ± 3.38	10.9 ± 5.03	8.69 ± 3.21	9.64 ± 4.10	0.271
Magnesium, mg/d	260 ± 101	309 ± 111	277 ± 101	274 ± 102	0.753
MUFA, g/d	21.3 ± 11.2	24.8 ± 11.3	21.3 ± 10.9	23.7 ± 10.3	0.773
PUFA, g/d	15.3 ± 8.12	17.7 ± 10.0	14.5 ± 7.59	16.3 ± 8.23	0.701
Niacin, mg/d	19.2 ± 6.92	25.9 ± 11.1	20.4 ± 8.03	21.6 ± 12.9	0.346
Omega3, g/d	0.33 ± 0.23	0.32 ± 0.24	0.17 ± 0.13	0.20 ± 0.13	< 0.001
Omega6, g/d	13.1 ± 7.73	14.8 ± 9.31	12.4 ± 6.98	13.9 ± 7.87	0.713
Riboflavin, µg/d	1.50 ± 0.62	1.88 ± 0.87	1.59 ± 0.63	1.83 ± 1.07	0.063
SFA, g/d	21.4 ± 9.43	25.6 ± 11.5	22.1 ± 11.1	24.4 ± 10.6	0.595
Selenium, mg/d	0.03 ± 0.02	0.05 ± 0.04	0.04 ± 0.03	0.04 ± 0.03	0.642
Thiamin, mg/d	1.68 ± 0.68	2.15 ± 0.91	1.74 ± 0.68	1.78 ± 1.30	0.938

*P* value less than 0.05 was considered significant

Values are based on mean ± standard deviation

^†^based on ANCOVA test adjusted for energy intake

*DII* Dietary inflammatory index, *RMR* Resting metabolic rate, *MUFA* Mono unsaturated fatty acids, *PUFA* Poly unsaturated fatty Acids, *SFA* Saturated fatty acids

The mean of VO_2Max_ (mL/kg/min) was lower in participants that were classified as high DII/low RMR compared to those in low DII/high RMR (*p* value = 0.02), this significant association was remained significant after controlling for confounders (*p* value = 0.04). Post hoc Tukey test revealed no significant differences between other categories in comparison with low DII/high RMR group. Participants with a high DII score and low RMR had lower VO_2Max_ (L/min) and VO_2Max_ (LBM) compared with those with low DII score and high RMR. However, the mean of VO_2max_ (L/min) and VO_2max_ (LBM) after adjustment for confounders, had no significant differences in any classification (Table 3).

**Table 3:** Mean of cardiorespiratory fitness by combined effect of dietary inflammatory index and resting metabolic rate

	Interaction of DII and RMR				*p* value*	*p* value†	Comparison group	Post hoc *p* value	*p* value‡	*R*^2^
	Low DII/high RMR(1)	Low DII/low RMR(2)	High DII/low RMR(3)	High DII/high RMR(4)						
VO_2max_ (ml/kg/min)	33.81 ± 8.12	33.51 ± 9.11	28.23 ± 6.00	29.03 ± 6.05	< 0.001	0.004	(1) versus (2) (1) versus (3) (1) versus (4)	0.21 0.02 0.12	0.04	0.49
VO_2max_ (L/min)	2.35 ± 0.74	2.24 ± 0.78	2.11 ± 0.66	2.23 ± 0.70	0.37	0.56	(1) versus (2) (1) versus (3) (1) versus (4)	0.59 0.03 0.29	0.14	0.04
VO_2max_ (LBM)	47.81 ± 8.79	47.72 ± 8.14	46.71 ± 8.35	46.82 ± 8.14	0.81	0.68	(1) versus (2) (1) versus (3) (1) versus (4)	0.48 0.04 0.22	0.89	0.03

*DII* Dietary inflammatory index, *RMR* Resting metabolic rate, *LBM* Lean body mass, *VO*_*2max*_ Maximal oxygen consumption, *R*^*2*^ R square

*p* value* obtained from one-way analysis of variance (ANOVA) was used to compare DII/RMR classifications

Values are based on mean ± standard deviation

*p* value less than 0.05 was considered significant

*p* value‡ Adjusted for age, sex, energy intake, smoking, physical activity and body mass index

*p* value† obtained from polynomial linear regression

*p* value‡ obtained from analysis of covariance (ANCOVA)

Multivariate adjusted odds ratios and 95% confidence intervals for CRF by the combined effect of DII and RMR are given in Table 4. In the crude model, those who were in the high DII/low RMR group, compared to the low DII/high RMR group, were less likely to have higher VO_2max_ (ml/kg/min) (OR 0.75; 95% CI 0.10, 0.85, *p *= 0.02); this association remained significant after adjusting for confounding variables (OR 0.72; 95% CI 0.18, 0.82, *p *= 0.04). Moreover, we found that participants in high DII/low RMR group, had lower odds of VO_2max_ (L/min) which was significant (OR 0.84, 95% CI 0.18, 0.89, *p *= 0.03). When potential confounders were taken into account, such association remained significant (OR 0.75, 95% CI 0.11, 0.89, *p *= 0.03). In the crude model, those who were in the high DII/low RMR group, compared to the low DII/high RMR group, were less likely to have higher VO_2max_ (LBM) (OR 0.85; 95% CI 0.05, 0.90, *p *= 0.01); this association remained significant after adjusting for confounding variables (OR 0.79; 95% CI 0.30, 0.92, *p *= 0.02). There was also no significant combined association of dietary inflammatory index and resting metabolic rate on cardiorespiratory fitness even after controlling for covariates.

**Table 4:** Odd ratios and 95% CIs for cardiorespiratory fitness by combined effect of dietary inflammatory index and resting metabolic rate

	Interaction of DII and RMR						
	Low DII/high RMR	Low DII/low RMR		High DII/low RMR		High DII/high RMR	
		OR (95% CI)	*P* value*	OR (95% CI)	*P* value*	OR (95% CI)	*P* value*
*VO*_*2max*_* (ml/kg/min)*							
Crude	1	0.92 (0.41, 1.52)	0.18	0.75 (0.10, 0.85)	0.02	1.05 (0.55, 1.65)	0.15
Model1	1	0.78 (0.29, 1.09)	0.12	0.83 (0.16, 0.97)	0.02	0.73 (0.28, 1.86)	0.20
Model2	1	0.76 (0.28, 1.07)	0.20	0.72 (0.18, 0.82)	0.04	0.81 (0.31, 2.11)	0.23
*VO*_*2max*_* (L/min)*							
Crude	1	1.47 (0.22, 1.58)	0.40	0.84 (0.18, 0.89)	0.03	0.78 (0.37, 1.65)	0.30
Model1	1	0.48 (0.20, 1.34)	0.37	0.81 (0.12, 0.88)	0.02	0.93 (0.40, 1.40)	0.32
Model2	1	0.46 (0.19, 1.20)	0.36	0.75 (0.11, 0.89)	0.03	0.97 (0.41, 1.35)	0.34
*VO*_*2max*_* (LBM)*							
Crude	1	1.46 (0.70, 1.84)	0.51	0.85 (0.05, 0.90)	0.01	1.56 (0.75, 1.64)	0.43
Model1	1	1.18 (0.51, 1.70)	0.34	0.81 (0.10, 0.94)	0.02	1.29 (0.57, 1.94)	0.45
Model2	1	1.13 (0.49, 1.61)	0.58	0.79 (0.30, 0.92)	0.02	1.26 (0.55, 1.90)	0.48

Data are presented as odds ratio (95% CI)

Cox and Snell R square for VO_2max_ (ml/kg/min) is 0.30, for VO_2max_ (L/min) is 0.04, and for VO_2max_ (LBM) is 0.04

*DII* Dietary inflammatory index, *RMR* Resting metabolic rate, *Model 1* Adjusted for age, sex, education status, smoking, *Model 2* Adjusted for age, sex, education status, smoking, physical activity

*Obtained by binary logistic regression and all the *P* values was compared to low DII/high RMR group as reference category

## Discussion

According to our cross-sectional study, the mean of VO_2Max_ (mL/kg/min), VO_2max_ (L/min) and VO_2max_ (LBM) was lower in participants that were classified as high DII/low RMR compared to those in low DII/high RMR. After controlling for covariates, those who were in the high DII/low RMR group, compared to the low DII/high RMR group were 28% less likely to have higher VO_2max_ (ml/kg/min). Moreover, we found that participants in high DII/low RMR group had 25% lower odds of VO_2max_ (L/min) which was significant. In the final model, those who were in the high DII/low RMR group, compared to the low DII/high RMR group were 21% less likely to have higher VO_2max_ (LBM).

In line with our results, a study by Potteiqer et al. [30] showed that participants lost 5 kg of body weight and about 4% of their adipose tissue during a 16-month exercise program. Also, after nine months, it was associated with a significant increase in VO_2max_ and a significant increase in RMR in both sexes. Eventually, the results showed that following a moderate-intensity aerobic exercise program along with reduced caloric intake from foods lead to an increased RMR and weight loss and body fat in obese people [30]. A cross-sectional study on apparently healthy adults with mean BMI equal to 25.6 kg/m^2^ showed that VO_2max_ is positively associated with RMR [31]. Moreover, this study revealed that those with VO_2max_ and lower RMR, had better body composition profiles including lower visceral fat, trunk fat, and body fat mass [31]. Moreover, a study conducted by Broeder and colleges on normal-to-overweight men failed to show any relationship between RMR and CRF [32]. On the other hand, positive stepwise gradient in RMR according to tertiles of CRF in a cross-sectional study by shook et al. indicate the key role of aerobic capacity on resting metabolic rate. In this study, participants with moderate to high CRF had higher RMR than those with low CRF [33]. Previous results by Kim and colleges have also shown that a difference in measured RMR and predicted RMR in obese men and also shown that there is a significant difference between measured RMR and predicted RMR in Korean obese men. This study also reported a positive association between their aerobic capacity and RMR [34]. Another study by Smith et al. showed there is no significant relationship between aerobic capacity and RMR in healthy women in the age range of 19 to 30 years [35]. In addition, Ormsbee et al. [36] showed that a period of 35–42 days of swim detraining such as light-moderate physical exercise after a competitive swim in healthy men and women leads to the following results: (a) 1.3%, 12.2% increase in weight and body fat, respectively. (b) 7.7% decrease in VO_2Max_, and (c) 7% decrease in RMR, without any change in blood lipids. It should be noted that increasing in body weight and specially body fat may cause the drop in CRF and RMR.

The highest quantity of oxygen that an individual may utilize when participating in intense or strenuous activity is known as VO2 max or maximal oxygen consumption. This measurement is generally considered the best indicator of cardiovascular fitness and aerobic endurance [37]. Payandeh et al. [37] show that higher adherence to a higher pro-inflammatory potential diet may be associated with less VO_2Max_ (ml/kg/min). In contrast, previous results from a case–control study by Scott et al. showed the DII score was associated with systemic inflammation increase and less lung function. They also reported that an increase by one unit of DII score can elevate the risk of asthma by 70% [38].

Contrarily, in Asia, Ren et al. found only a slight association between the DII and the prevalence of the metabolic syndrome components (with the exception of blood pressure) among adults in eight Chinese cities [39]. Similarly, in a study conducted among the Lebanese population [40] and in the Fasa Cohort Study (FACS) [41] conducted in Iran, no significant association was reported between the DII and the prevalence of Metabolic syndrome. Furthermore, Asadi et al.'s study of a middle-aged Iranian population revealed no association between the DII and total cardiovascular disease, myocardial infarction, stable angina, or unstable angina [42]. Result of an umbrella review of meta-analyses of observational studies indicated that adherence to a diet with high inflammatory index might be associated with a higher risk of colorectal cancer, cardiovascular disease, and all-cause mortality [20]. The reasons for these conflicting findings may be related to the various sample sizes of studies or also various studies design, even though lack of adjustment for different confounders such as individuals medical and family history.

Two possible mechanisms mentioned in studies regarding the effect of physical activity on RMR are as follows: physical activity can affect RMR by accelerating muscle growth and affecting physiological processes. Cardiorespiratory fitness also appears to be a key predictor of RMR, although it operates independently of skeletal muscle mass [43]. This difference in RMR according to CRF groups is probably due to physiological processes [33]. Other mechanisms for explaining how CRF and physical activity affect RMR levels may be related to sympathetic nervous system regulations [44–46], the function of neuroendocrine system [47, 48], structure changing of myocytes [49], and various immune responses [50].

Several limitations are better to be considered in the explanation of our findings. The main limitation of our study is its cross-sectional design which does not accurately state the cause-and-effect relationship. Another limitation is the low sample size of our study. Also, we calculated DII based on 29 dietary items and data regarding 16 dietary items were not available in this study. However, some strengths of our study should be noted that the present study is the first study from Iran to examine the combined association of dietary inflammatory index and RMR on cardiorespiratory fitness. As well, we have used the standardized 168 items FFQ that has been collected for the Iranian eating habits assessment. Moreover, we adjusted several important confounders which could affect our main results. Therefore, the results of the present study can be a positive step in the direction of anti-inflammatory diet recommendations by physicians.

## Conclusion

In conclusion, consumption of a pro-inflammatory diet in combination with low RMR status is associated with 28% lesser odds of having better CRF compared with those with anti-inflammatory diet with high RMR among Iranian healthy men and women. In other words, we have observed the importance of physical activity and how the inflammatory index can influence it. However, more studies on this area are needed to confirm the veracity of our results. This study suggests that researchers should focus on dietary indexes rather than single antioxidant nutrients for having a better judgment.

## Data Availability

The datasets generated or analyzed during the current study are not publicly available due to restrictions, e.g., their containing information that could compromise the privacy of research participants but are available from the corresponding author on reasonable request.

## Abbreviations

DII: Dietary inflammatory index
BMI: Body mass index
FFQ: Food frequency questionnaire
RMR: Resting metabolic rate
ANOVA: Analysis of variance
ANCOVA: Analysis of covariance
MUFA: Monounsaturated fatty acids
PUFA: Polyunsaturated fatty acids
WHtR: Waist-to-height ratio
CRF: Cardiorespiratory fitness
WC: Waist circumference
FM: Fat mass
FFM: Fat free mass
LBM: Lean body mass
CRP: C-reactive protein
CVD: Cardiovascular disease
VO_2Max_: Maximum (max) rate (V) of oxygen (O_2_) consumption
CLA: Conjugated linoleic acid
